# Event and Comment

**Published:** 1908-04-15

**Authors:** 


// DENTAL REGISTER.

Vol. LXII. April 15, 1908. No. 4.

### EVENT AND COMMENT.

### Prescription Writing.

We frequently in dental society discussions hear some one deprecate the practice among dentists of recommending proprietary remedies instead of writing prescriptions for their patients. There can be no question but this is a very common practice even with the very best practitioners. The venders of various antiseptic lotions, for instance, keep the profession supplied with sufficient free samples to keep them continually reminded of their remedies, and dentists find it so much easier to simply tell their patients to call at the drug store and get a bottle of such a remedy, than to stop and write a prescription for the case in hand. It may also be said, if the whole truth were told, that a considerable percentage of our practitioners could not write a prescription that would be compatible pharmaceutically or which would be as well put up and as palatable as are many of the so called proprietary preparations. Our pharmacists will not take the pains to compound a formula requiring time without considerable more expense than would be involved should one of the trade preparations be used, which are prepared with great care and precision and on a scale that makes it not only profitable to the manufacturer, but produces a much more acceptable preparation pharmaceutically, than can ordinarily be produced by temporary pharmacy. Another practical difficulty which will need to be met in any such effort, and it is one that can hardly be overcome, is that the expense to the patient will be so much more that we are liable to be accused of reprehensible business methods. A physician ordinarily will not write a prescription for less than a dollar, and a dentist can not afford to do it for less, and it is not likely that any prescription we should feel called upon to compound would be filled by the pharmacist for less than fifty cents. If then we should prescribe a simple four ounce mouth lotion of boric acid and lanin in water it will cost our patient one dollar and fifty cents. While we can send them to the drug store for a similar preparation and the same amount much better prepared at a total cost to them of twenty-five cents. This is a day when the work of the pharmacist has changed to a dispensing rather than a compounding function. Elixirs and tablets have taken the place of individual prescribing and compounding. Experts report their favorite formulae to the manufacturing chemist who willingly supplies the market with the most carefully compounded product in the most acceptable form for the patient. The physician in place of writing a prescription with as many as twelve or eighteen ingredients, now simply writes Smith's Compound Cathartic Pills, XX, and directions for administration to patient. Or what is more commonly done he carries about with him a medicine case containing all the drugs he ordinarily requires, in compact form, from which he supplies his patient at the bedside. In the article by Dr. Stern in this issue he cites as an illustration of the seeming fraud of the manufacturer, the case of a mouth wash which he purchased from his druggist, and which according to the label contained only alcohol, formalin and Acetate of Amyl, and of course water, and which cost him 25 cents for a three ounce bottle, and he finds it could be made for forty cents for three fourths of a gallon. When we look at the matter in this way it does seem as though we were being imposed upon by the manufacturers. But we have no doubt Dr. Stern or his patients would much rather pay the twenty-five cents for the trade remedy than a less amount for one that was compounded simply on the basis of the formula as given on that label; make it up and see how you would like such a preparation for a steady diet. The manufacturer must and does have to make his preparations efficient and agreeable, and this in many cases requires skill, and facilities to produce that deserve more than average compensation. The manufacturers of the new breakfast foods have discovered that absolute cleanliness in the manufacture and handling of their products is essential to success and they spare neither men nor facilities to attain this object. The cracker maker now puts his goods in antiseptic wrappings to get them to the consumer in the most palatable and hygienic condition. Even the millers wash and sterilize the wheat before they grind it into flour. And so it is that our medicines are today being prepared by reliable manufacturers with all the care and skill that science and good business methods can suggest or command. Then why should we waste our time and spend our patients money on expedients of doubtful value just for the sake of seeming professional. We do not write these words to weaken any one's loyalty to his calling or to take from it one iota of its dignity. Sentiment is one of the strongest forces for keeping intact our self-respect, and we would be very sorry to lower any one's ideals built upon the prevalent sentiment that our standing as a profession is dependent on our loyalty to the traditions of history. We would not cut loose from our traditions which may seem in conflict with our present environment, but believe it the part of good judgment as well as tending to increase our capacity for broader as well as more efficient service that we adjust our ideals to existing conditions. We shall make little progress by broadly condemning or failing to utilize the equipment that modern skill and science has placed in our way. It will be far better for us to sift out of the mass of commercial remedies that are being brought to our attention through these sources, and see if there be any good in them. If we are sufficiently skillful and devoted to our calling this should be an important part of our work. If there is any good in any of them we are the best fitted to find it out. And should any be found lacking it should be our duty to make known our finding to the profession, as no one can do this alone, and some are so careless or indolent that they make no well directed efforts. Let us be broad and charitable enough to recognize the work of merit and equally earnest in condemning the false and unworthy.

We commend most cordially the two articles in this issue on Dental Therapeutics to the careful perusal and earnest consideration of our readers.

**The Oldest Dental Journal.**

Dr. Nelville S. Hoff,

Esteemed Friend:—It may be presumption, it may be "crass" ignorance, or it may be dense stupidity, that prompts this communication. Looking up the past of the Dental Register, I find on page 51, Jan. 15, 1906, the statement that the Dental Register is the "second oldest dental journal extant."

It began October 1847, and is still going, always has been going, just as steady as a yankee clock. Now, I want to know, did any dental journal now published begin sooner, and keep going as the Register has done?—Not that we know of.—Editor Register.

I want to know; my grand-dad was born about 1770, my dad was born 1793, he had the same name as my grand-dad. I was born—well never mind when, my name is about the same as theirs was, now I want to know, does that make me in this year of Grace 1908, one hundred and thirty eight years old, and the oldest man in all these United States?

Is every John Smith now living as old as the first John Smith on record would be if now living?

Three dental journals published in the United States have been named the "American Journal of Dental Science." So far as the records show, no connection of any kind or character has existed between these separate publications. They had nothing in common but the name. Neither the second nor third, can anymore claim to be the oldest dental journal in the world, than I can claim to be the oldest dentist, because I bear my grand-dad's name.

The Dental Register can truthfully place on its title page "The Oldest Dental Journal in the World," it is the only dental journal in the world that can.

Yours truly,

WILLIAM H. TRUEMAN.

No. 900 Spruce St., Philadelphia, Pa.,

March 12, 1908.

